# Day-to-Day Boundary Fluctuations in Coronal Holes: Causes and Consequences

**DOI:** 10.1007/s11207-026-02610-8

**Published:** 2026-02-02

**Authors:** I. Ugarte-Urra, Y.-M. Wang, K. Muglach, N. R. Sheeley

**Affiliations:** 1 Space Science Division, Naval Research Laboratory, Washington, DC 20375 USA; 2 Code 674, NASA Goddard Space Flight Center, Greenbelt, MD 20771 USA; 3 Catholic University of America, Washington, DC 20064 USA; 4 Visiting Research Scientist, Lunar and Planetary Laboratory, University of Arizona, Tucson, AZ 85721 USA

## Abstract

Extreme-ultraviolet (EUV) images from the Atmospheric Imaging Assembly on the Solar Dynamics Observatory and the EUV Imager on the Solar Terrestrial Relations Observatory show that coronal hole boundaries often change from one day to the next on spatial scales up to several supergranules. Such changes may occur even in the absence of nearby sunspots or transient activity. We attribute the fluctuations to the action of supergranular convection, which continually rearranges the photospheric flux distribution both near and far from the hole boundaries. The boundary displacements may exceed a supergranular diameter because, in addition to simple advection, the open magnetic flux may undergo interchange reconnection with the long closed loops rooted just outside the boundary. This injects streamer material into the heliospheric plasma sheet but does not lead to a mixing of open and closed flux, whose interface remains clearly defined in EUV images and qualitatively consistent with current-free extrapolations of the (instantaneous) photospheric field. However, the boundary fluctuations are likely to be a major cause of the well-known variability of the slow solar wind, with the footpoint locations of the wind intercepted by a given spacecraft continually changing relative to the hole boundary on timescales of a day or less. This variability reflects the steep increase in the rate of flux-tube divergence toward the boundary, which leads to rapid changes in the measured wind speeds and densities. We also describe an unusual case in which a long-lived coronal hole forms suddenly without any nearby flux emergence, apparently as a result of transient-driven interchange reconnection with the north polar hole.

**Supplementary Information:**

The online version contains supplementary material available at 10.1007/s11207-026-02610-8.

## Introduction

Coronal holes contain “open” magnetic fields along which plasma escapes into the heliosphere to form the solar wind; because their densities are generally lower than in the closed corona, they appear dark in extreme-ultraviolet (EUV) and X-ray images (for a general review, see Cranmer 2009). They are located inside “unipolar” areas dominated by a single magnetic polarity, which form as active regions (ARs) decay and their flux spreads over the solar surface due to supergranular convection, differential rotation, and meridional flow. In the absence of a sufficient amount of opposite-polarity flux in the surrounding areas, field lines rooted in the unipolar region may extend to heights where the thermal pressure overcomes the restraining magnetic tension. It is important to recognize that the properties, formation, and evolution of coronal holes depend not only on the directly underlying photospheric field, but also on the distribution of flux far outside the hole boundaries (cf. Heinemann et al. 2020, who found no correlation between the area evolution of coronal holes and their signed flux densities).

As reviewed in Wang (2009), the polar fields and polar coronal holes owe their existence to the tendency for ARs to emerge with their eastward, trailing-polarity sectors located somewhat poleward of their leading-polarity sectors (Joy’s law). Because some of the leading-polarity flux diffuses across the equator and cancels with its opposite-hemisphere counterpart, the surface meridional flow preferentially transports trailing-polarity flux toward the poles, canceling the old-cycle polar fields near solar maximum and establishing the new-cycle polar fields and holes. At the same time, the combined action of rotational shearing and supergranular diffusion acts to annihilate the nonaxiymmetric (longitudinally varying) component of the large-scale photospheric field on the flow timescale ($\tau _{ \mathrm{flow}}\sim R_{\odot}/v_{\mathrm{m}}\sim 2$ yr, assuming a poleward flow speed of $v_{\mathrm{m}}\sim 10$ m s^−1^). However, unless all sunspot activity ceases for an extended period ($\gtrsim \tau _{\mathrm{flow}}$), a purely axisymmetric state will not be reached even at solar minimum.

In this article, we use EUV images and magnetograms from the Solar Dynamics Observatory (SDO) and EUV observations from the Solar Terrestrial Relations Observatory (STEREO) A to show that coronal holes and their boundaries continue to undergo significant day-to-day changes even during periods of very low sunspot activity, such as 2019 – 2020. We attribute these variations to the continued presence of nonuniformities in the large-scale photospheric flux and polarity distribution, combined with the stochastic effects of supergranular convection. We also suggest that such short-term fluctuations in coronal hole boundaries (which occur at all phases of the solar cycle) are a major cause of the variability of the slow solar wind.

## Formation of Coronal Holes Following the Emergence of ARs

ARs are the original source of the unipolar regions inside which coronal holes form. The total amount of open flux on the Sun, $\Phi _{\mathrm{open}}$, depends mainly on its total dipole ($l = 1$) strength, with the quadrupole ($l = 2$) component also contributing significantly when the polar fields undergo their reversal at solar maximum. A new AR will increase (decrease) $\Phi _{\mathrm{open}}$ if it emerges in such a way as to increase (decrease) the strength of these lowest-order multipoles. Even if there is little net change in $\Phi _{\mathrm{open}}$, however, there may be a redistribution of open flux in the vicinity of the new AR due to “interchange” reconnection between open and closed field lines.

The approximate locations of open flux can be derived by applying a potential-field source-surface (PFSS) extrapolation to the global photospheric field. The current-free coronal field $\boldsymbol{B}$, which we express as a sum of spherical harmonics up to multipole $l = 31$, is constrained to be radial at a heliocentric distance $r = R_{\mathrm{ss}}$; at the inner boundary $r = R_{\odot}$, $B_{r}$ is matched to the photospheric flux distribution (see Wang and Sheeley 1992). All field lines that reach the source surface, here taken to be at 2.5 $R_{\odot}$, are considered to be open, and their footpoint areas are predicted to coincide with coronal holes.

**Figure 1:**
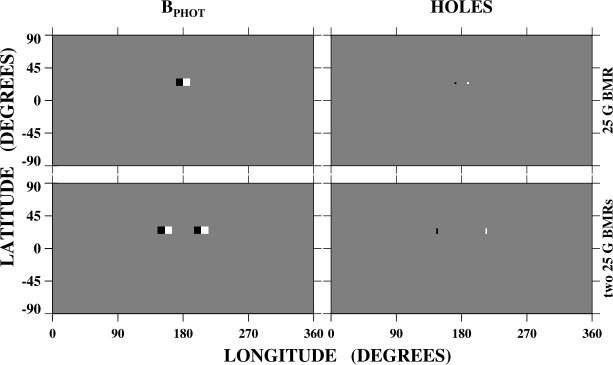
Top row: Photospheric field (left) and open field regions (right) associated with a single BMR centered at latitude $L = +25^{\circ}$. The bipole has positive (white) leading polarity, uniform strength 25 G, longitudinal (latitudinal) extent 20^∘^ (10^∘^), and no axial tilt. The background photospheric field has been set to zero. Small areas of open flux appear at opposite ends of the BMR. Bottom row: Same as top row, but for two BMRs centered 50^∘^ apart in longitude. In this case, larger areas of open flux appear at the easternmost and westernmost ends of the system, but not in the region between the two BMRs. The locations of open flux were determined by applying a PFSS extrapolation and tracing downward from the source surface at $r = 2.5$ $R_{\odot}$.

To illustrate the effect of new ARs on the distribution of open flux, we use the PFSS model to derive the open field areas associated with idealized bipolar magnetic regions (BMRs). The latitude–longitude maps in Figure 1 show the photospheric field (left panels) and distribution of open flux (right panels) corresponding to a single BMR (top row) and a pair of identical BMRs separated by 50^∘^ in longitude (bottom row). Each BMR, assigned a uniform strength of 25 G, is centered at latitude $L = +25^{\circ}$ and has a total longitudinal (latitudinal) extent of 20^∘^ (10^∘^), with positive leading/westward polarity and no axial tilt. Small areas of open flux may be seen at the eastern- and westernmost ends of the single BMR (top right panel). These are the footpoints of the field lines that extend highest into the corona and cross the source surface. In the case of the two side-by-side BMRs, the open flux appears at the easternmost end of the eastern BMR and the westernmost end of the western BMR (bottom right panel). The facing sectors of the BMRs connect to each other and remain closed. The open field areas associated with the two-BMR system are much larger and contain three times as much flux as those associated with the single BMR, because the equatorial dipole ($l=1$, $\vert m\vert =1$) strength is almost twice as large and because of the presence of a strong ($l=2$, $\vert m\vert =2$) quadrupole component.

**Figure 2:**
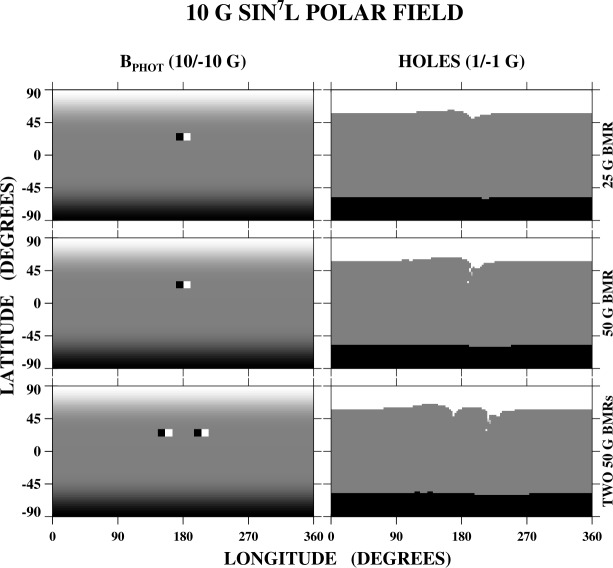
Interaction between a BMR and a polar field of the form $B_{r} = 10$ G $\sin ^{7}L$, characteristic of solar minimum conditions. Top row: Photospheric field (left) and open field regions (right) for the case of a bipole of uniform strength 25 G centered at $L = +25^{\circ}$. Again, as in Figure 1, the BMR has positive leading polarity, longitudinal (latitudinal) extent 20^∘^ (10^∘^), and no axial tilt. The gray scale for the photospheric field (open flux) is saturated at ± 10 G (± 1 G). Some of the polar hole flux adjacent to the negative-polarity sector of the BMR closes down, while a small equatorward spur forms longitudinally adjacent to the positive-polarity sector. Middle row: As in the top row, except that the strength of the BMR has been increased from 25 to 50 G. The spur of the north polar hole increases in size and extends to lower latitudes, and a small area of open flux now appears within the positive-polarity sector of the BMR itself. Bottom row: Here, two 50 G BMRs separated by 50^∘^ in longitude have been deposited at $L = 25^{\circ}$. This results in the formation of two equatorward extensions of the north polar hole, with the smaller one directed toward the positive-polarity sector of the eastern BMR and the larger one extending toward the positive-polarity sector of the western BMR, with a small area of open flux appearing at the edge of the BMR itself.

We now consider the effect of depositing a BMR in a background photospheric field of the form 10 G $\sin ^{7}L$, which is representative of solar minimum conditions (see Svalgaard, Duvall, and Scherrer 1978; Petrie and Patrikeeva 2009; Wang, Robbrecht, and Sheeley 2009). As before, the BMR(s) are centered at latitude $L = +25^{\circ}$ and have total longitudinal (latitudinal) extents of 20^∘^ (10^∘^). The top row of Figure 2 shows the case of a BMR with a uniform strength of 25 G. The poleward-concentrated background field gives rise to open field areas (polar holes) extending to just below latitude 60^∘^ in each hemisphere. The BMR acts to distort the boundary of the nearby positive-polarity polar hole and to transfer some of its open flux to lower latitudes. Because the emergence of the bipole barely changes the total dipole strength, which is dominated by its axial ($l=1$, $m=0$) component, the total open flux remains almost unchanged and the main effect is to redistribute the open flux, presumably via interchange reconnection. Here, we may also think of the BMR as acting to tilt the Sun’s dipole axis, with the tilt angle depending on the strength of the equatorial dipole component of the BMR relative to the background axial dipole. In the middle row of Figure 2, the strength of the BMR has been increased from 25 to 50 G. The north polar-hole boundary becomes even more distorted; a lobe with a narrow equatorward extension forms and a small area of open flux appears at the western extremity of the BMR itself.

**Figure 3:**
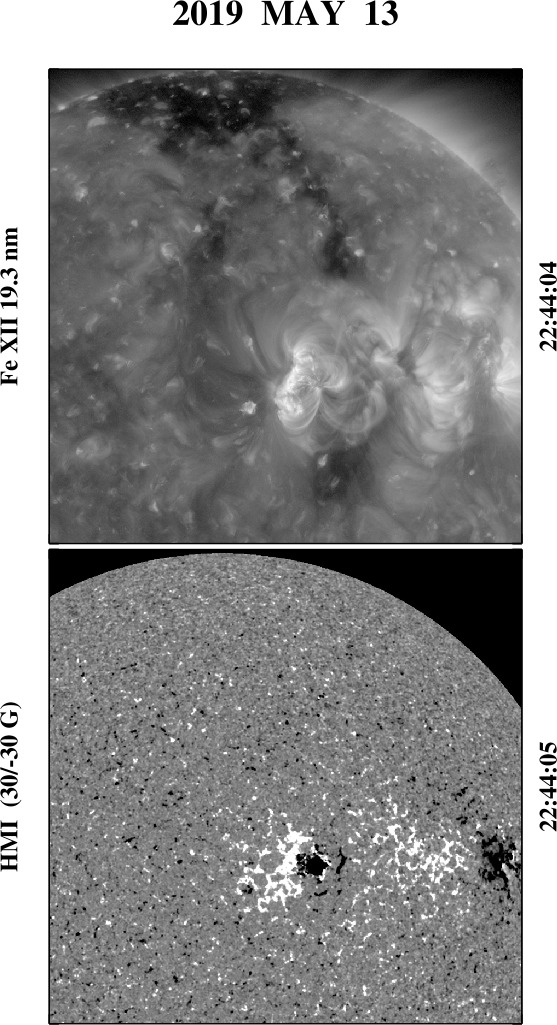
An example of twin polar-hole extensions. Top panel: SDO/AIA Fe xii 19.3 nm image recorded on 2019 May 13. Bottom panel: Corresponding SDO/HMI line-of-sight magnetogram, saturated at ± 30 G. The two ARs situated just north of the equator are NOAA 12740 (to the west) and 12741 (to the east). A wind stream with peak speed ≈ 600 km s^−1^ and outward interplanetary magnetic field (IMF) polarity was observed near Earth around May 16.

In the bottom panels of Figure 2, two 50 G BMRs centered 50^∘^ apart in longitude have been deposited at $L = +25^{\circ}$. A pair of equatorward extensions form, directed toward the positive-polarity sector of each BMR; a small area of strong open flux also appears just inside the edge of the westernmost BMR. An example of such a configuration may be seen in the Atmospheric Imaging Assembly (AIA) Fe xii 19.3 nm image in the top panel of Figure 3, recorded on 2019 May 13; the corresponding Helioseismic and Magnetic Imager (HMI) line-of-sight magnetogram is displayed in the bottom panel. The north polar region has positive polarity, and the two equatorward extensions are directed toward the positive-polarity sectors of the large ARs NOAA 12740 and 12741, which are located just north of the equator. These ARs have negative leading polarity, opposite to that of the BMRs in Figure 2 but in accordance with Hale’s law for the northern hemisphere during Solar Cycle 24.

The interchange reconnection inferred from Figure 2 involves the closing-down of polar hole flux longitudinally adjacent to the negative-polarity sector of the BMR(s) and the simultaneous opening-up of background flux adjacent to the positive-polarity sector. Conceptually, we may think of the long closed loops overlying the positive-polarity sector as being pushed outward and then exchanging footpoints with the north polar-hole field lines. When the BMR is sufficiently strong (bottom two rows of Figure 2), its outermost loops will also encounter and undergo footpoint exchanges with the overlying polar-hole field lines; this accounts for the small area of open flux appearing at the western edge of the single BMR (second row of Figure 2) and at the westernmost end of the two-BMR system (bottom row).

We remark that the polar hole extensions that form following the emergence of ARs tend to rotate at the rate of the ARs themselves, not of the underlying photospheric plasma. This quasi-rigid rotation is maintained by continual interchange reconnection between the open field lines along the boundaries of the extensions and the neighboring closed loops, with the reconnection occurring at the streamer cusps/null points (see Wang and Sheeley 2004).

**Figure 4:**
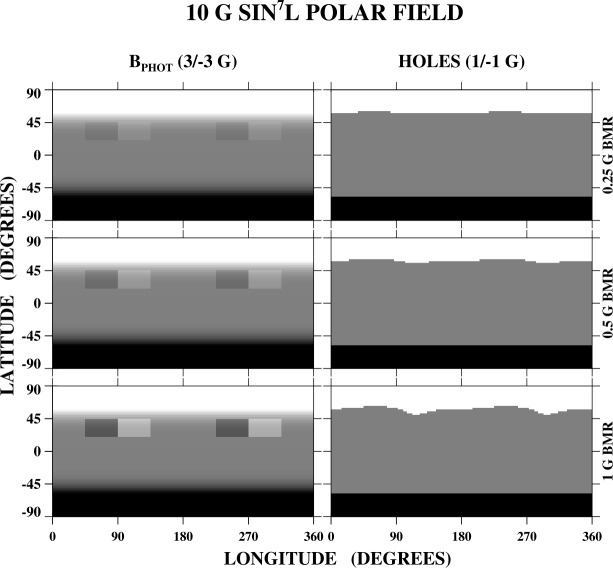
Even weak large-scale fields, such as those associated with long-decayed ARs, may warp the polar hole boundaries. For illustrative purposes, we show the effect of two large, very weak BMRs on the polar holes, where the polar fields are again of the form 10 G $\sin ^{7}L$. The BMRs are centered at $L = +25^{\circ}$ and have longitudinal (latitudinal) extents of 90^∘^ ($25^{\circ}$). They have a uniform strength of 0.25 G (top row), 0.5 G (middle row), and 1 G (bottom row). Gray scale for the photospheric field (left panels) is saturated at ± 3 G, while that for the open flux (right panels) is saturated at ± 1 G. The amplitude of the corrugations in the north polar-hole boundary increases as the BMR strength increases, but even a large-scale field as weak as 0.25 G has an effect on the nearby boundary.

As ARs decay, their flux spreads over the solar surface, resulting in non-uniformities in the large-scale photospheric field which, in the absence of new AR emergence, may take as long as ≈ 2 yr to decay away. Even if the decaying fields are weak compared with the polar fields, they may significantly warp the polar hole boundaries. Figure 4 shows the effect of two large, very weak BMRs on the neighboring polar-hole boundary, where we have again assumed a background field of the form 10 G $\sin ^{7}L$. Each BMR extends from $L = +25^{\circ}$ to $L = +40^{\circ}$ and has a total longitudinal width of 90^∘^. The strengths of the two BMRs are 0.25 G (top row), 0.5 G (middle row), and 1 G (bottom row). The corrugations of the polar hole boundary become larger as the BMR strength increases, but are present even when the strength is as low as 0.25 G. In reality, the AR remnants will be rotationally sheared as they are transported to higher latitudes.

## EUV Observations of Coronal-Hole Boundary Changes

In this and the following sections, we present examples of rapid changes in coronal holes and their boundaries, using mainly observations from SDO/AIA and HMI taken during 2019 – 2020, close to the last sunspot minimum. To help guide the eye, we have plotted color contour lines on the images to indicate the approximate locations of the hole boundaries. These contours were determined by applying a 15-pixel smoothing to the Fe xii 19.3 nm images (to reduce the small-scale noise) and adopting 55 DN s^−1^ as the count level separating coronal holes from the background; the latter value was chosen to agree with our own visual estimates of the boundary locations. The actual boundaries will differ in detail because of projection effects and the presence of intervening material in the line of sight, limb brightening, and the fact that not all dark regions are coronal holes (as illustrated by Figure 14 below); such effects cannot be accounted for by the use of a single intensity (or intensity ratio) threshold. To show changes, we also superpose the boundary contours determined for a given day on the 19.3 nm image recorded 24 hr later, after correcting for photospheric differential rotation using the rot_xy.pro routine provided by the IDL SolarSoft library; because the rotation of the boundary may deviate from that of the underlying plasma, the correction represents an approximation.

**Figure 5:**
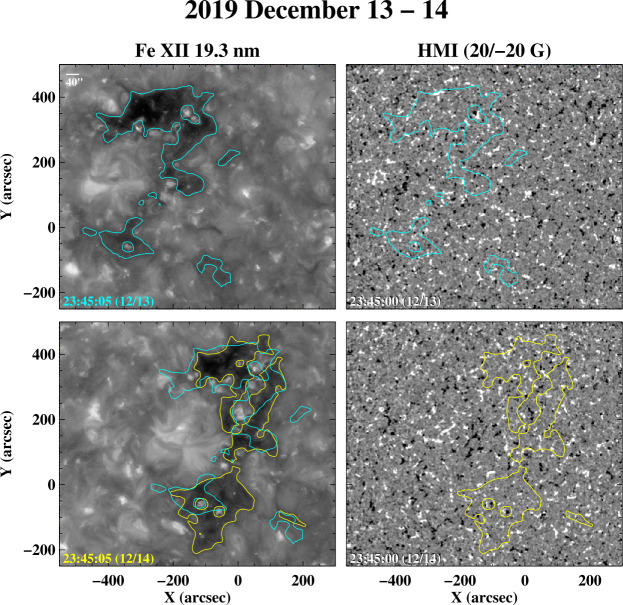
AIA Fe xii 19.3 nm images and HMI magnetograms showing a transequatorial coronal hole on 2019 December 13 (top row) and 24 hr later on December 14 (bottom row). Here and in subsequent figures, the color contours indicate the approximate locations of the hole boundaries; the boundaries from a given day are also overplotted on the 193 nm image recorded the next day after correcting for differential rotation. The white horizontal bar in the top left panel indicates the approximate diameter (40^′′^) of a supergranular cell. The boundaries of the positive-polarity hole shown here undergo significant changes (especially in the south) between December 13 and 14, despite the absence of any large-scale flux emergence on the disk. This hole is the remnant of a large equatorial hole that formed during the decay of the two ARs seen in Figure 3, and which was the source of a high-speed stream that persisted in the ecliptic (for seven rotations) through the second half of 2019. At this late stage, the ambient positive- and negative-polarity flux elements are increasingly well mixed. An animation of the evolution of this coronal hole is available. The video shows superposed Fe xii 19.3 nm images and HMI magnetograms recorded at ≈ 15 minute intervals between ≈ 23:45 UT on December 13 and ≈ 23:45 UT on December 14.)

The decay of the pair of large ARs seen in Figure 3 led to the formation of a large equatorial coronal hole that persisted until the end of 2019 and was the source of a recurrent high-speed wind stream in the ecliptic, as indicated by near-Earth OMNI data.^1^ The Fe xii 19.3 nm images in Figure 5 show the remnant of this long-lived hole as it rotated toward central meridian on 2019 December 13 – 14; the corresponding HMI magnetograms are on the right. The hole has positive polarity and partially surrounds a decayed bipole that emerged on the Sun’s farside. Although the Sun is very quiet at this time, the boundaries of the hole undergo significant changes during the 24 hr period separating the two 19.3 nm images. Some of the changes substantially exceed a supergranular diameter (≈ 25 – 30 Mm or ≈ 34^′′^ – 41^′′^), which is roughly the width of one of the “coronal cells” representing the bases of the Fe xii loops (Sheeley and Warren 2012).^2^ This relatively narrow hole was the source of a wind stream with peak speeds of just over 500 km s^−1^ recorded near Earth on December 18 – 19.

**Figure 6:**
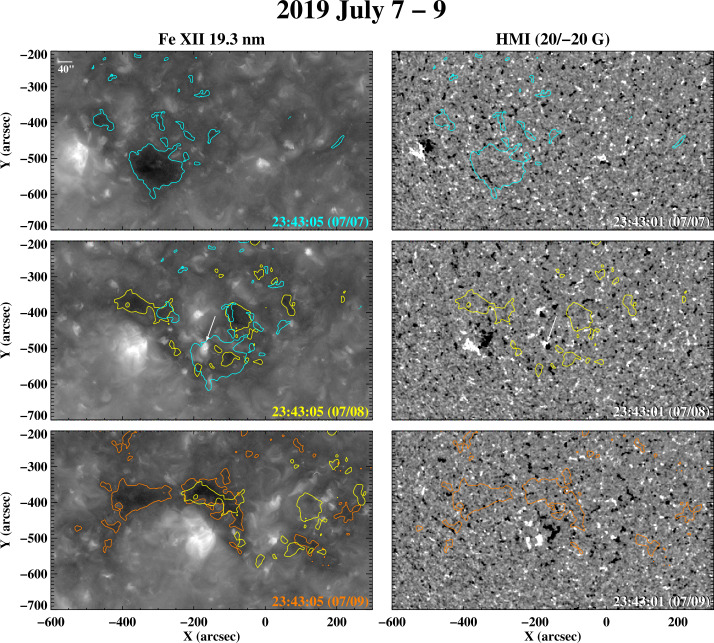
Fe xii 19.3 nm images and HMI magnetograms recorded during 2019 July 7 – 9, showing a negative-polarity hole that formed around NOAA 12744, located at $L\approx -27^{\circ}$. The hole undergoes significant evolution over this two-day period, as the small AR decays and spreads, the surrounding supergranular network is reshuffled, and bright points (one of which is marked by an arrow) emerge on July 8 near or within the hole.

Figure 6 shows the day-to-day evolution of the negative-polarity coronal hole(s) that formed around a small AR (NOAA 12744) which emerged at latitude $L\approx -27^{\circ}$ on 2019 July 6. The emergence of a pair of bright points/small bipoles to the west of the AR on July 8 shifts the boundaries of and partially closes down the new hole seen to the southwest of the AR on July 7. In this case, the boundary evolution appears to be the result both of the reshuffling of the network by supergranular convection (on a ≈ 1 – 1.5 day timescale, corresponding to the lifetime of an individual supergranule; see Del Moro et al. 2004; Hirzberger et al. 2008; Roudier et al. 2014), which may in turn drive interchange reconnection, and the injection of a small amount of opposite-polarity flux by ephemeral regions.

**Figure 7:**
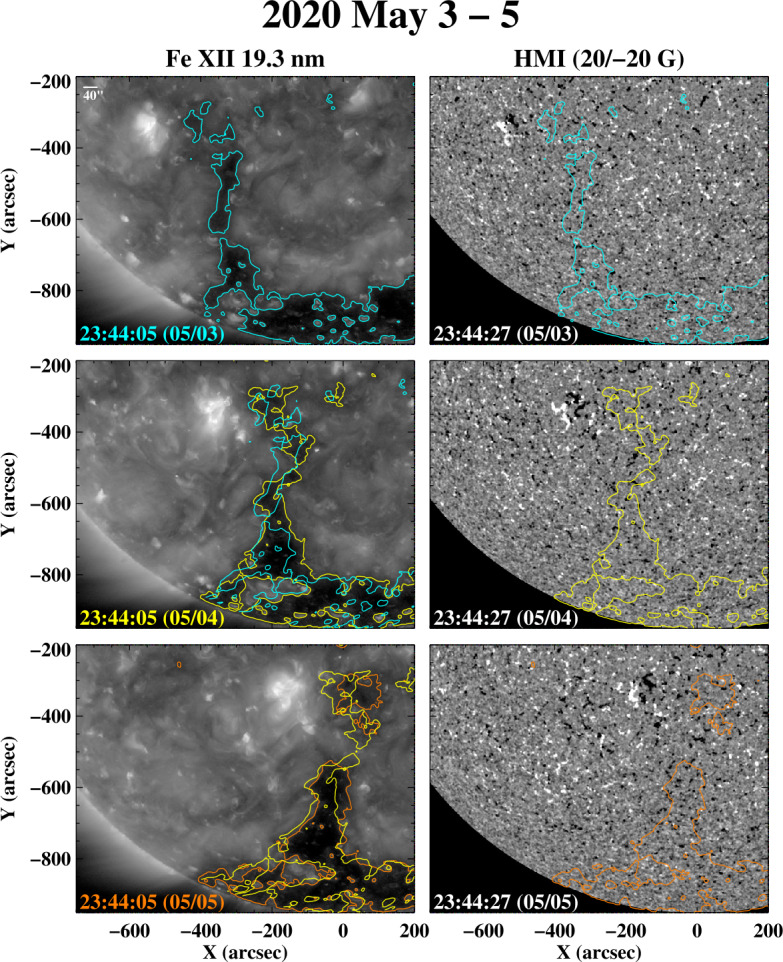
Evolution of an equatorward extension of the south polar hole during 2020 May 3 – 5. The negative-polarity extension has formed to the west of a small AR located at latitude $L\approx -24^{\circ}$. The boundaries of the hole are outlined by the adjacent Fe xii 19.3 nm loop structures, whose bases have dimensions on the order of a supergranular diameter.

The sequence of 19.3 nm and HMI images in Figure 7 shows a small AR that emerged at $L\approx -24^{\circ}$ on 2020 May 2 and the equatorward extension of the south polar hole that formed (or re-formed) to its west. The shape and width of the negative-polarity extension undergo noticeable day-to-day changes during May 3 – 5. This narrow spur was the source of low-speed (≈ 300 – 340 km s^−1^) wind at Earth after May 11, as indicated by OMNI data and PFSS extrapolations.

## Fluctuations in Polar Hole Boundaries

We now describe four examples of short-term changes in the polar hole boundaries, again as seen in AIA Fe xii 19.3 nm images and HMI line-of-sight magnetograms.

**Figure 8:**
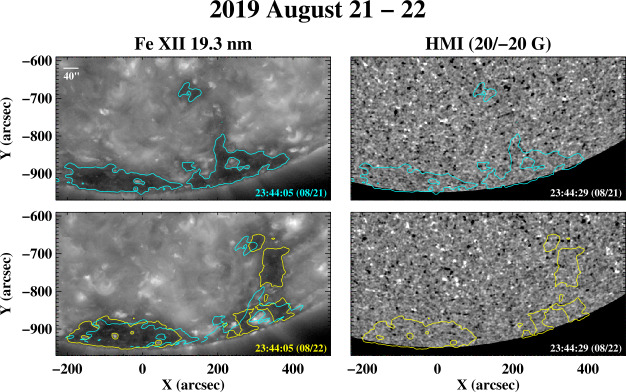
Evolution of the boundary of the negative-polarity south polar hole, 2019 August 21 – 22. Again, color contours indicate the approximate location of the boundary; in the 19.3 nm image recorded on August 22, the boundary contour from the previous day has been superposed after correcting for differential rotation. Despite the absence of any large-scale activity on the disk, the equatorward spur widens significantly over this 24 hr period.

Figure 8 shows the negative-polarity south polar hole during 2019 August 21 – 22. The narrow equatorward spur seen on August 21 widens considerably over the next 24 hr, despite the absence of any large-scale activity or flux emergence on the disk. The change occurs on a spatial scale of a few supergranular diameters. As in the case of the lower-latitude coronal holes, the boundary fluctuations may be attributed not just to simple advection, but also to interchange reconnection between open and closed flux along the boundary, as their footpoints undergo random displacements in the supergranular flow field. The location and amplitude of the fluctuations also depend on the distribution of photospheric flux both near and far from the boundary.

**Figure 9:**
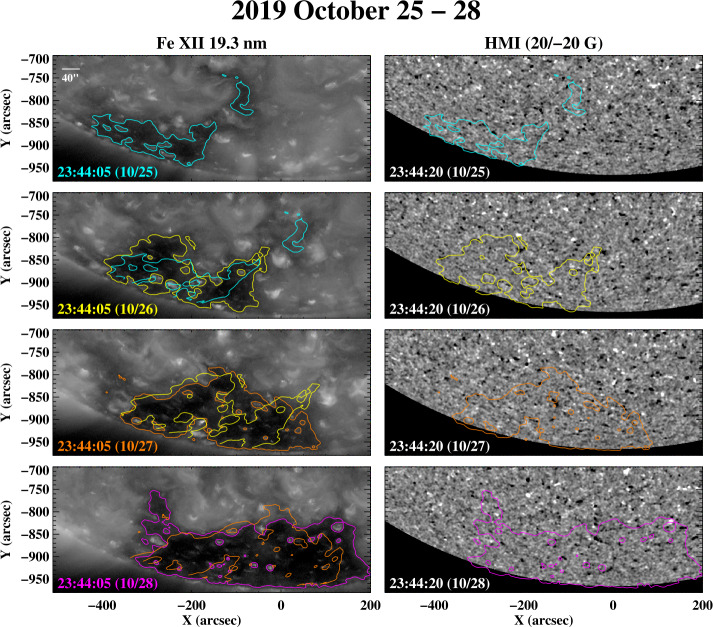
Day-to-day changes in the corrugations of the south polar-hole boundary, 2019 October 25 – 28. Again, the solar disk is very quiet at this time.

Figure 9 shows the evolution of the south polar-hole boundary during 2019 October 25 – 28, close to the minimum in the 13-month smoothed sunspot number reached in 2019 December. Even allowing for line-of-sight projection and limb brightening effects, the boundary undergoes pronounced daily variations.

**Figure 10:**
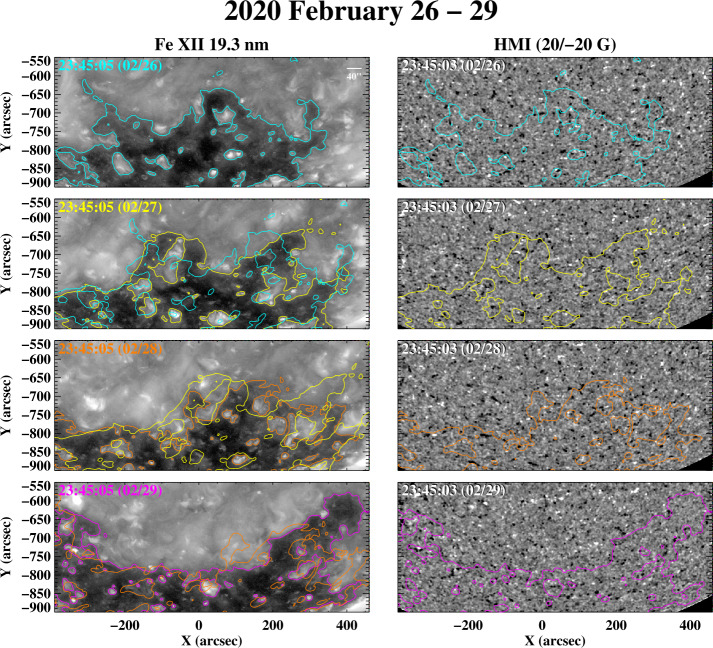
The south polar-hole boundary observed during 2020 February 26 – 29, just after the solar activity minimum. Again, the corrugations and equatorward extensions continually fluctuate from one day to the next, on spatial scales of up to several supergranular diameters. Nevertheless, the location of the boundary generally remains well defined.

The sequence in Figure 10 displays the day-to-day fluctuations of the south polar-hole boundary during 2020 February 26 – 29. Especially striking is the rapid growth of the equatorward extension, by several supergranular diameters, between February 28 and 29. The near-Earth wind speeds associated with this boundary region ranged from ≈ 400 to over 500 km s^−1^, with the peak speeds being recorded late on February 29.

**Figure 11:**
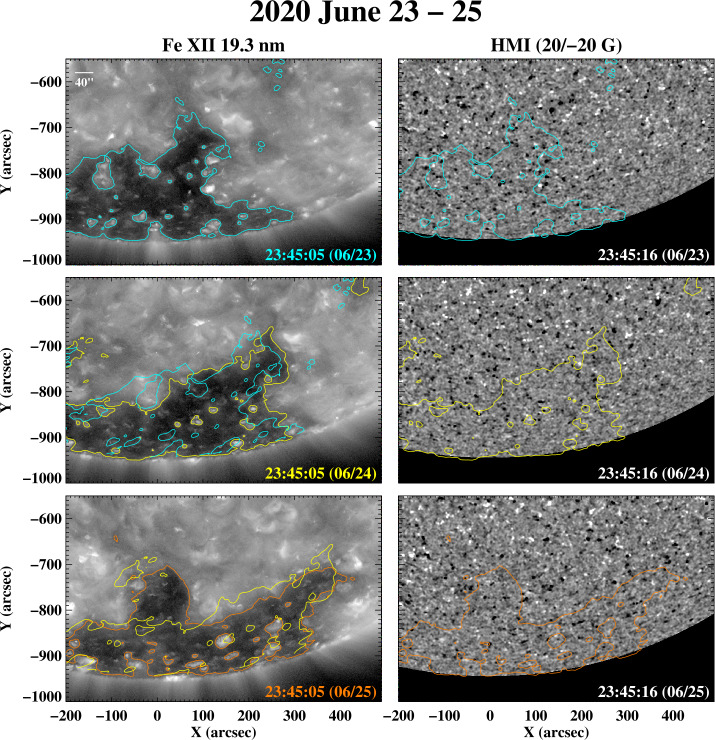
Changes in the south polar-hole boundary during 2020 June 23 – 25. Note the formation of a new equatorward spur or protrusion on June 25, with a latitudinal extent of ≈ 130^′′^ or ≈ 3.3 supergranular diameters, and a longitudinal width of ≈ 115^′′^ or ≈ 2.9 supergranular diameters.

**Figure 12:**
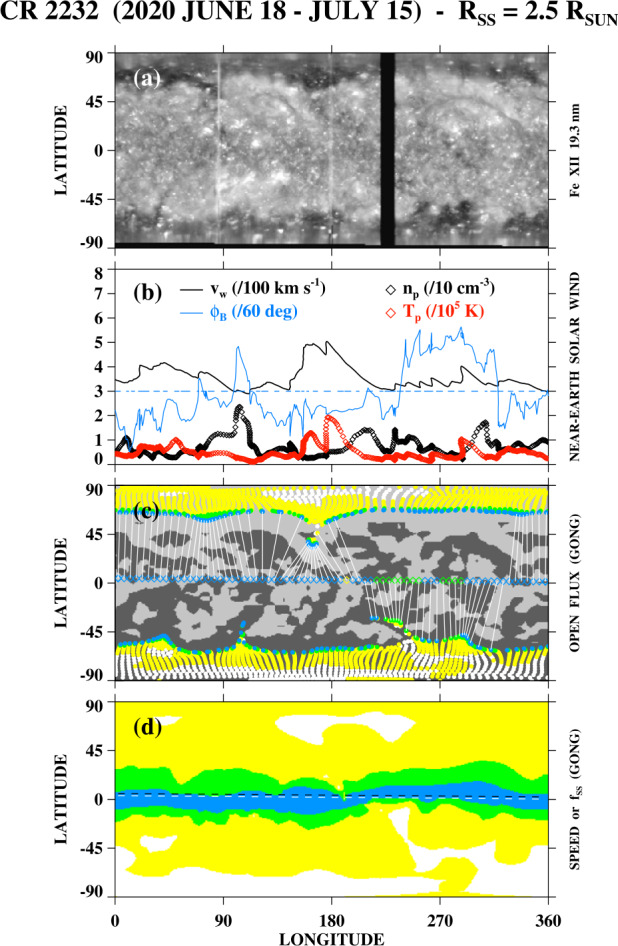
Latitude–longitude maps and near-Earth solar wind measurements for CR 2232 (2020 June 18 to July 15). (a) Distribution of 19.3 nm emission from SDO/AIA. (b) OMNI measurements of the proton bulk speed $v_{\mathrm{w}}$, proton density $n_{\mathrm{p}}$, proton temperature $T_{\mathrm{p}}$, and IMF longitude angle $\phi _{B}$. Horizontal dashed line marks $\phi _{B} = 180^{\circ}$; $\phi _{B} > 180^{\circ}$ ($\phi _{B} < 180^{\circ}$) corresponds to inward (outward) IMF. The data, plotted in time-reversed order, have been smoothed by taking 10 hr running averages and mapped back to longitude at the source surface assuming a transit time $\propto v_{\mathrm{w}}^{-1}$. (c) Open field regions at the solar surface, derived from a PFSS extrapolation of a GONG photospheric field map and color-coded by the local value of the flux-tube expansion factor $f_{\mathrm{ss}} = (R_{\odot}/R_{\mathrm{ss}})^{2}(B_{0}/B_{\mathrm{ss}})$ or, equivalently, the predicted wind speed. Blue: $f_{\mathrm{ss}} > 30$ ($v_{\mathrm{w}} < 450$ km s^−1^). Green: $11 < f_{\mathrm{ss}} < 30$ (450 km s$^{-1} < v_{\mathrm{w}} < 550$ km s^−1^). Yellow: $4 < f_{\mathrm{ss}} < 11$ (550 km s$^{-1} < v_{ \mathrm{w}} < 650$ km s^−1^). White: $f_{\mathrm{ss}} < 4$ ($v_{\mathrm{w}} > 650$ km s^−1^). Dark (light) gray background denotes $B_{r} < 0$ ($B_{r} > 0$). Horizontal row of diamonds (similarly color-coded and plotted at 5^∘^ intervals of longitude) indicates the expansion factors of flux tubes directed into the ecliptic plane, with white lines connecting their ecliptic positions to their photospheric footpoints. (d) Distribution of expansion factors $f_{\mathrm{ss}}$ at $r = R_{\mathrm{ss}} = 2.5$ $R_{\odot}$ or, equivalently, of predicted wind speeds $v_{\mathrm{w}}$ at 1 au, as derived from the GONG photospheric field measurements. Horizontal dashes mark the latitude of the ecliptic. The south polar-hole boundary seen in Figure 11 is located near longitude $\phi \approx 270^{\circ}$ and is the source of near-Earth wind with speeds in the range ≈ 310 – 410 km s^−1^.

As a final example of polar-hole boundary changes during very quiet times, the Fe xii 19.3 nm images and magnetograms in Figure 11 show the south polar hole during 2020 June 23 – 25. Again, the boundary fluctuations occur on a scale of a few (≈ 3) supergranular diameters. To provide a more global perspective, Figure 12a shows a latitude–longitude map of 19.3 nm emission during Carrington rotation (CR) 2232 (2020 June 18 to July 15), assembled from a time-reversed sequence of individual AIA full-disk images; the polar-hole boundary corrugations seen in Figure 11 are located near Carrington longitude $\phi \approx 270^{\circ}$. Note that the map does not show the instantaneous shape of the fluctuating boundary, but represents a composite in which each point along the boundary is observed around the time of its central meridian passage. In Figure 12b, we display OMNI measurements of the proton bulk speed $v_{\mathrm{w}}$, proton density $n_{\mathrm{p}}$, proton temperature $T_{\mathrm{p}}$, and interplanetary magnetic field (IMF) longitude angle $\phi _{B}$. The horizontal dashed line marks $\phi _{B} = 180^{\circ}$, $\phi _{B} > 180^{\circ}$ ($\phi _{B} < 180^{ \circ}$) corresponds to inward (outward) IMF. The data, plotted in time-reversed order, have been smoothed by taking 10 hr running averages and mapped back to the Carrington frame by assuming a Sun–Earth transit time $\propto v_{\mathrm{w}}^{-1}$ and taking into account the longitude shift due to solar rotation.

In the bottom two panels of Figure 12, we have applied a PFSS extrapolation to a Global Oscillation Network Group (GONG) photospheric field map for CR 2232 to determine the distribution of open flux at the solar surface (Figure 12c) and to predict the associated wind speeds (Figure 12d).^3^ Here, the footpoints of open field lines were determined by tracing downward from the source surface $r = R_{\mathrm{ss}} = 2.5$ $R_{\odot}$, and each flux tube was assigned an expansion factor, defined by

1 $$ f_{\mathrm{ss}} = \left (\frac{R_{\odot}}{R_{\mathrm{ss}}}\right )^{2} \left ( \frac{B_{0}}{B_{\mathrm{ss}}}\right ), $$

where $B_{0}$ denotes the footpoint field strength and $B_{\mathrm{ss}}$ is the field strength at the source surface. The value of $f_{\mathrm{ss}}$, which measures the areal expansion of a flux tube between $r = R_{\odot}$ and $r = R_{\mathrm{ss}}$, has been shown to be inversely correlated with the solar wind speed $v_{\mathrm{w}}$ at 1 au (Levine, Altschuler, and Harvey 1977; Wang and Sheeley 1990; Bravo and Stewart 1997; Arge and Pizzo 2000; Poduval 2016; Majumdar et al. 2025). The footpoint areas and distribution of expansion factors at the source surface are color-coded as follows (see Wang and Sheeley 2025): blue ($v_{\mathrm{w}} < 450$ km s^−1^, $f_{\mathrm{ss}} > 30$); green (450 km s$^{-1} < v_{\mathrm{w}} < 550$ km s^−1^, $11 < f_{\mathrm{ss}} < 30$); yellow (550 km s$^{-1} < v_{\mathrm{w}} < 650$ km s^−1^, $4 < f_{\mathrm{ss}} < 11$); white ($v_{\mathrm{w}} > 650$ km s^−1^, $f_{\mathrm{ss}} < 4$). The colored diamonds in Figure 12c indicate the expansion factors/predicted speeds associated with flux tubes that are directed into the ecliptic plane, with the white lines connecting them to their footpoints; light (dark) gray background denotes $B_{r} > 0$ ($B_{r} < 0$). The horizontal dashed line in Figure 12d marks the latitude of the ecliptic.

From Figure 12, we conclude that the boundary corrugations of the south polar hole, seen in the Fe xii 19.3 nm map near longitude 270^∘^, are the source of near-Earth wind with speeds in the range ≈ 310 – 410 km s^−1^. The PFSS extrapolations show that flux tubes with large expansion factors (coded blue and green) are rooted along this boundary region and connect to the ecliptic plane. As expected, the IMF is directed inward ($\phi _{B} > 180^{\circ}$), matching the negative polarity of the footpoint photospheric field. Note also the fluctuations in $n_{\mathrm{p}}$ within this sector, with the densities rising near the sector boundaries (Figure 12b). Unfortunately, without a plasma monitor that corotates with the Sun, we are unable to determine the wind speed and density variations associated with a fixed Carrington longitude (rather than with a succession of different longitudes) and to relate them directly to the 19.3 nm boundary fluctuations at that longitude.

## Background Coronal Holes and Coronal Voids

Many small coronal holes live for less than a solar rotation, and (as shown by Inglis et al. 2019) some even have lifetimes significantly less than a disk passage. At sunspot maximum, the frequent emergence of ARs continually transfers open flux from one location to another via interchange reconnection (see Wang et al. 2024). Near sunspot minimum, the continual reshuffling of decayed AR flux by supergranular convection may result in the formation of short-lived “background” holes whose shapes and boundaries change from day to day.

**Figure 13:**
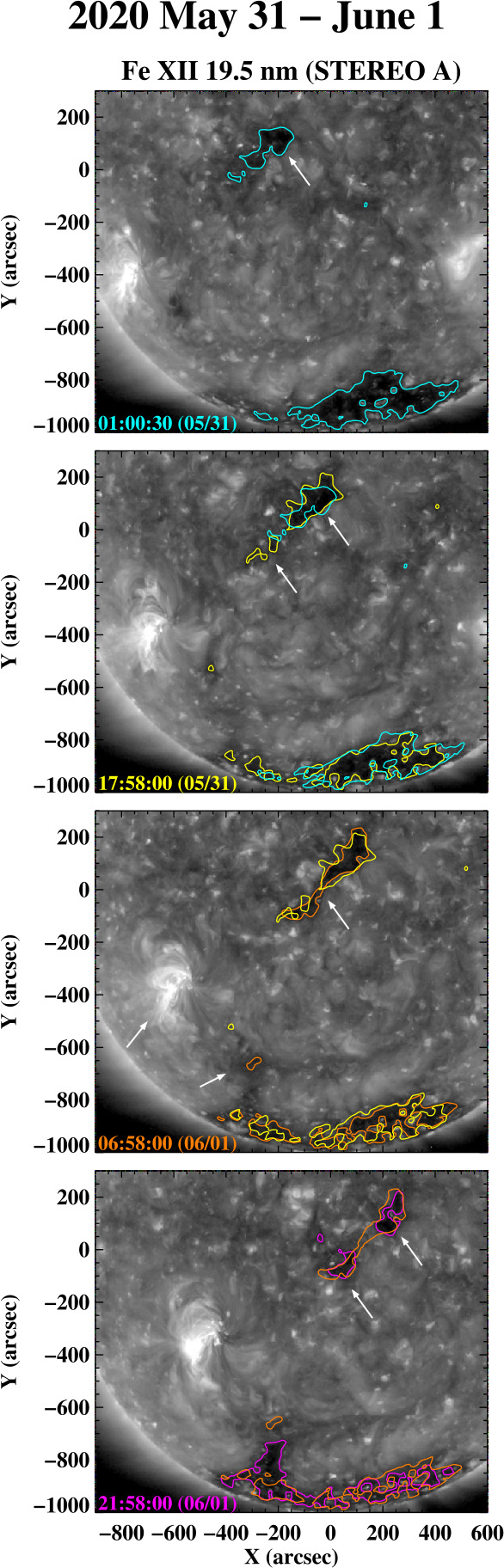
Sequence of STEREO/EUVI A Fe xii 19.5 nm images showing the evolution of an elongated equatorial coronal hole during 2020 May 31 to June 1. Here, the boundary contours are based on a count level of 16 DN s^−1^ separating coronal holes from the background. The hole, which has positive polarity according to subsequently recorded HMI magnetograms, is situated inside a quiet background region. To the southeast, an equatorward extension of the negative-polarity south polar hole forms on the west side of AR NOAA 12765.

Figure 13 displays a sequence of Fe xii 19.5 nm images recorded by the EUV Instrument (EUVI) on STEREO A during 2020 May 31 to June 1. At 01:00 UT on May 31 (top panel), a small “background” hole may be seen near the equator; this hole is located in a positive-polarity region (as indicated by subsequently recorded HMI magnetograms) and formed on May 29. Later on May 31, a second small hole appears to the southeast (second panel), and the two holes merge on June 1 (third panel). The resulting narrow, elongated hole then splits again later on the same day (bottom panel). Although not shown here, the southernmost hole disappears on June 3 and the equatorward remnant fades into the background on June 5.

Figure 13 also shows an AR (subsequently designated NOAA 12765) rotating past the east limb at latitude $L\approx -24^{\circ}$, and an associated equatorward extension of the south polar hole forming on June 1 (third panel). As viewed from SDO, this equatorward extension, located at Carrington longitude $\phi \approx 115^{\circ}$ (CR 2231), crossed central meridian on June 8 – 9; however, OMNI data show the near-Earth solar wind from this longitude having outward instead of inward IMF polarity, with speeds of just over 300 km s^−1^. This is consistent with PFSS extrapolations, which predict that Earth was connected to rapidly expanding flux tubes rooted at the edge of the north polar hole at this time.

**Figure 14:**
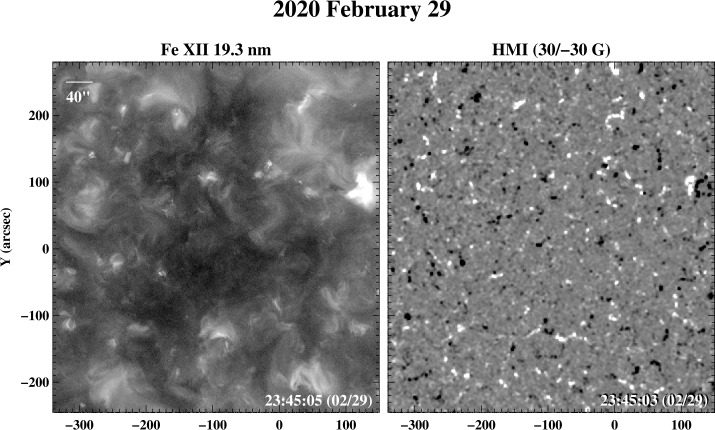
Near solar minimum, EUV images often show dark areas on the disk that overlie very weak, mixed polarity fields and that are not coronal holes. This shows an example of a “coronal void” recorded at disk center on 2020 February 29. Left: AIA Fe xii 19.3 nm image. Right: HMI magnetogram saturated at ± 30 G.

Near sunspot minimum, much of the solar surface outside the polar regions is occupied by mixed-polarity “quiet Sun” fields. Areas where the network is especially weak appear dark in EUV images and may easily be mistaken for coronal holes. Nölke et al. (2023) describe examples of such “coronal voids” observed in the 17.4 nm channel by Solar Orbiter on 2021 February 23. The AIA 19.3 nm image and HMI magnetogram in Figure 14 show a dark area located near disk center on 2020 February 29. The photospheric field is seen to be very weak in the darkest parts of the region. From the absence of a clearly dominant polarity, we deduce that we are observing a closed field region with reduced coronal heating, rather than a coronal hole. This conclusion also agrees with PFSS extrapolations.

## Formation of a Long-Lived Coronal Hole Following a Transient Event

**Figure 15:**
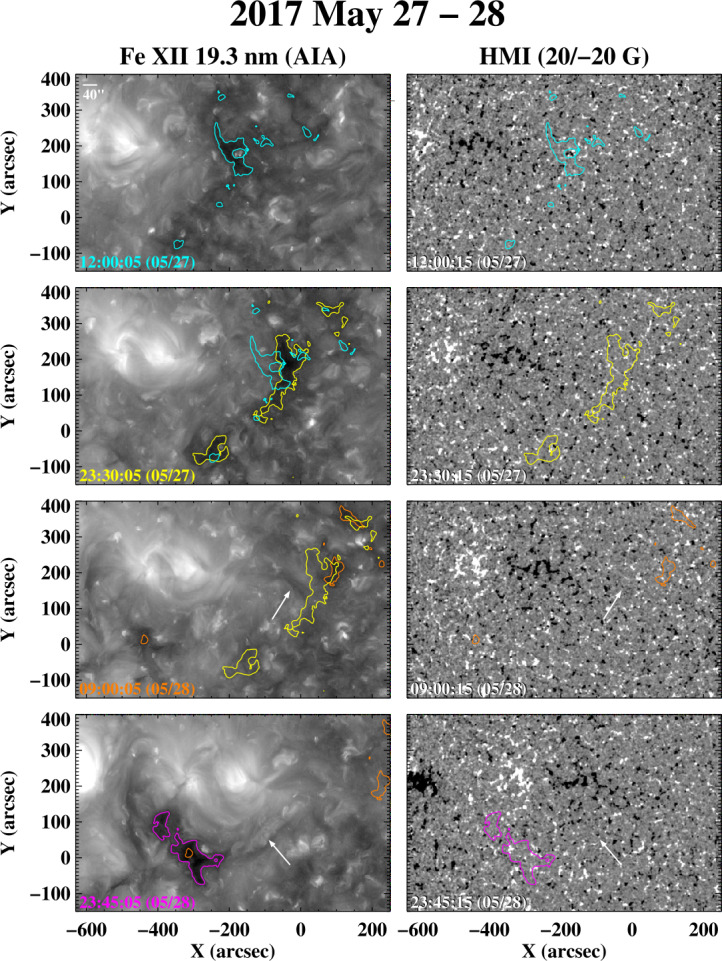
Formation of a non-transient coronal hole following one or more transient events in the vicinity of a decayed AR, 2017 May 27 – 28. In the Fe xii 19.3 nm images recorded on May 27 and early May 28, a V-shaped front preceded by a dark band of open flux is seen propagating westward from the AR remnant. The wave appears to originate from the PIL separating the negative-polarity sector of the remnant from the surrounding positive-polarity background region. The arrow in the 09:00 UT image points to a piece of dark filament material near the PIL; the new coronal hole is located in the positive-polarity region on the south side of the AR remnant. The arrow in the 19.3 nm image taken at 23:45 UT on May 28 marks what appear to be small post-eruption loops along the PIL just to the west of the new hole, which remains visible as it rotates across the disk over the following week. An animation of this event is available. The video shows successive full-disk Fe xii 19.3 nm images recorded at ≈ 15 minute intervals between 01:10 UT on May 27 and 15:56 UT on May 30. The equatorial coronal hole is seen forming on May 28 and continuing to evolve over the next two days.

The sequence of Fe xii 19.3 nm images and HMI magnetograms in Figure 15, recorded during 2017 May 27 – 28, shows an unusual case in which a non-transient coronal hole forms suddenly after one or more transient events. The new equatorial hole has positive polarity and is located just south of a decayed AR (see the bottom two rows of Figure 15). A day earlier, on May 27, a V-shaped front is seen propagating westward from the negative-polarity sector of the remnant AR, preceded by a narrow dark area representing open flux that closes down again after the front passes. The front may have been triggered by a filament eruption along the polarity inversion line (PIL) separating the negative-polarity sector from the positive-polarity region on its west and northwest sides; a piece of dark filament material (arrowed) may be seen in the 19.3 nm image recorded at 09:00 UT on May 28. A second filament eruption may have occurred along the south side of the negative-polarity region, as suggested by the PIL-aligned cluster of compact loops (arrowed) in the 19.3 nm image in the bottom row of Figure 15. The new hole is located just to the east of this cluster, and has grown in size since it first appeared early on May 28.

Near-Earth OMNI data show a wind stream with peak speeds of 470 – 480 km s^−1^ on June 3 – 4, whose outward IMF polarity matches the polarity of the equatorial hole.

**Figure 16:**
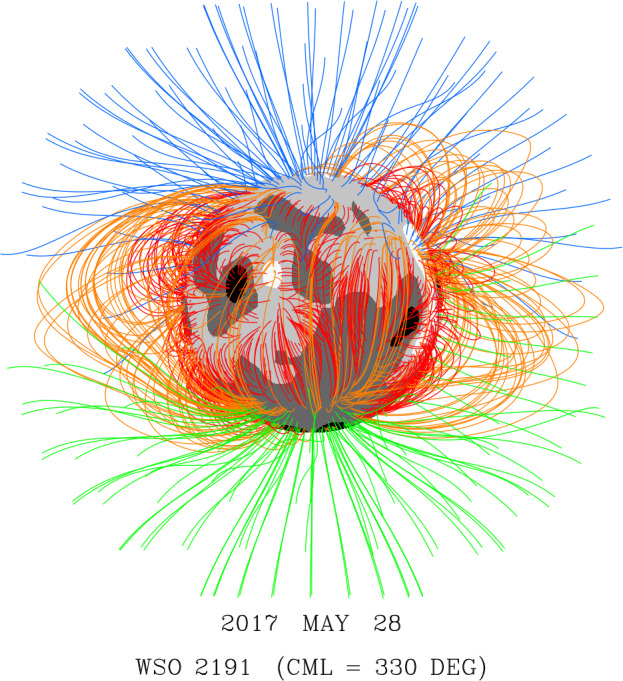
Coronal field-line configuration as viewed from Earth on 2017 May 28, derived by applying a PFSS extrapolation to WSO photospheric field measurements for CR 2191. Blue (green) denotes outward- (inward-) directed open field lines; closed loops are orange if they extend above $r = 1.5$ $R_{\odot}$, red otherwise. Gray-scale contours for the photospheric field are as follows: black ($B_{r} < -5$ G); dark gray (−5 G $< B_{r} < 0$); light gray ($0 < B_{r} < +5$ G); white ($B_{r} > +10$ G). The extrapolation does not show the coronal hole that forms on May 28 (Figure 15) in the positive-polarity region just below disk center. Instead, we note the presence of long loops that connect this unipolar area surrounding the AR remnant to the south polar region.

PFSS extrapolations of GONG, HMI, and Wilcox Solar Observatory (WSO) photospheric field maps for CR 2191 (2017 May 26 to June 23) do not predict an open field region at the location of the new coronal hole. Figure 16 shows the coronal field-line configuration derived from the WSO photospheric measurements. Here, central meridian is at Carrington longitude $\phi = 330^{\circ}$; the positive-polarity region in question lies close to disk center, with the negative-polarity sector directly northward of it. Although no open flux appears at this location, it is linked by long closed loops to the south polar region. We conjecture that the filament channel eruptions may have rearranged the overlying field in such a way as to drive interchange reconnection between these loops and the open field lines rooted at the edge of the north polar hole. This would have transferred positive-polarity open flux to the equatorial region and created new closed loops connecting the boundary of the north polar hole to the south polar region. Similarly, the transient opening-up of flux preceding the V-shaped front may have been driven by interchange reconnection between the long background loops linked to the south polar region and the north polar-hole field lines, with the process being reversed after the passage of the wave.

The failure of the PFSS model to predict the new equatorial hole might be due to the omission of currents induced by transient events, such as filament eruptions triggered by flux cancellation at the PIL. We also note that the presence of the filament channel itself implies the breakdown of the current-free assumption in its vicinity.

Hofmeister et al. (2025) have described another example in which a long-lived coronal hole formed following a filament eruption. In their event, which occurred on 2014 June 25, the new hole appeared to migrate westward (presumably as a result of interchange reconnection) and merge with a larger background hole.

## Summary and Discussion

It is well known that coronal holes may undergo rapid changes when ARs emerge and inject new photospheric flux (for more case studies, see Sheeley, Wang, and Harvey 1989; Wang et al. 2010). The present study shows that coronal hole boundaries routinely undergo substantial fluctuations, on scales of up to several supergranular diameters, even in the absence of nearby large-scale flux emergence. These fluctuations occur in coronal holes that form around decayed ARs, in background holes located in the quiet Sun, and along the polar hole boundaries. They are driven by supergranular convection, which continually rearranges the local network on daily timescales, by interchange reconnection between open field lines and long closed loops rooted near the hole boundaries, whose footpoints continually undergo random displacements in the supergranular flow field, and sometimes by the emergence of large ephemeral regions. These processes do not lead to a large-scale mixing of open flux and long closed loops, but the hole boundaries generally remain well defined.

Heinemann et al. (2023) have described a case in which a roughly north–south oriented coronal hole rapidly took on an east–west slant as it crossed central meridian during 2021 April 29 to May 1. In agreement with the present study, they attributed this change to the effect of interchange reconnection (which, as here, was likely to be driven by supergranular motions acting on the background photospheric flux distribution, not by rotational shearing).

Mason and Uritsky (2022) have developed a correlation technique for quantifying coronal-hole boundary variations on timescales much less than an hour, with the fluctuations (on spatial scales of ≈ 5 – 20 Mm) being attributed to interchange reconnection. They found systematic differences between the short-term fluctuations occurring at boundaries adjacent to pseudostreamers and those adjacent to helmet streamers. Although differences in the orientation or viewing angle of the Fe xii 19.3 nm structures along the boundary might also play a role, their results may be consistent with the tendency for pseudostreamers, which are confined by open flux of the same polarity, to be more stable than helmet streamers, which are surrounded by weak fields and show a tendency to continually expand outward.

On large scales, supergranular convection acts as a diffusive process directed away from flux concentrations such as those associated with ARs and the polar fields. The average equilibrium position of the polar hole boundaries near solar minimum is determined by a balance between the poleward meridional flow and the equatorward-directed diffusion from the concentrated “topknot” polar fields. However, on spatiotemporal scales comparable to individual supergranular cells and their lifetimes, the displacements are stochastic and resemble a random walk. This makes the day-to-day fluctuations in coronal hole boundaries very hard to predict, even using advective/flux transport models (see, e.g., Hickmann et al. 2015).

The empirical inverse correlation between the rate of coronal flux-tube expansion and the wind speed at 1 au predicts that flux tubes rooted just inside the boundaries of coronal holes and within small holes are sources of low-speed wind. The continual day-to-day fluctuations of the boundaries may thus be a major source of the well-known spatiotemporal variability of the slow solar wind, which contrasts with the relatively smooth and homogeneous velocity, temperature, density, and compositional structure characterizing the interiors of high-speed streams. In the PFSS model, $f_{\mathrm{ss}}\propto B_{\mathrm{ss}}^{-1}$ changes rapidly just inside the polar hole boundaries, with the largest values of $f_{\mathrm{ss}}$ and lowest asymptotic wind speeds (≈ 300 km s^−1^) being associated with open field lines rooted along the boundary itself. At any given longitude, the latitudinal distance between the boundary and the ecliptic will vary from day to day. By the same token, we would expect the footpoint of a flux tube that is connected to (e.g.) Earth to be located at varying distances from the boundary on such timescales, leading to corresponding fluctuations in the wind speed at Earth. Large density fluctuations may also occur because of the steep increase in $n_{p}$ near the heliospheric plasma sheet, which represents the interplanetary extension of the hole boundary.

The interchange reconnection between the open field lines rooted at the polar hole boundary itself and the neighboring helmet-streamer loops will act to inject streamer material into the heliospheric plasma/current sheet, which consists of narrow, raylike structures when seen in sharpened images (see Wang et al. 2007). It should be noted that the heliospheric plasma sheet is only a few degrees wide when viewed edge-on (see Figure 1 in Wang et al. 1998). This again suggests that the interactions between open field lines and streamer loops are confined to a relatively narrow boundary layer, instead of occurring over a broad region as advocated in some models for the origin of the slow solar wind (see, e.g., Rappazzo et al. 2012; Pontin and Wyper 2015; Higginson et al. 2017). Aslanyan et al. (2022) have modeled the dynamical structure of hole boundaries adjacent to helmet streamers and pseudostreamers; in their 3D MHD simulations, however, the interchange reconnection is driven by vortical motions with speeds of up to ≈ 10 km s^−1^, which is over an order of magnitude greater than the observed supergranular motions.

Finally, we emphasize that the interchange reconnection occurring along hole boundaries, which is localized at the streamer cusps and involves footpoint exchanges between open field lines and large-scale coronal loops, is fundamentally different from the interchange reconnection occurring in the interiors of coronal holes, which involves interactions between open field lines and small-scale loops or ephemeral regions. Recent observations of ubiquitous “jetlets” inside coronal holes suggest that such reconnection events may be responsible for heating coronal holes and energizing both the fast solar wind and most of the slow wind (see, e.g., Chitta et al. 2023, 2025; Raouafi et al. 2023). This footpoint reconnection is driven both by granular motions and by supergranular convection, giving rise to ohmic heating and chromospheric evaporation, as well as to Alfvén waves with periods ranging from minutes to hours (Wang 2020). Along rapidly expanding flux tubes, magnetic energy dissipation is confined to lower heights, resulting in larger mass flux densities but less energy per proton and slower wind. Along more slowly expanding flux tubes, the Alfvén waves propagate to greater distances before being reflected and dissipated, leading to higher wind speeds.

## Supplementary Information

Below are the links to the electronic supplementary material.

(MP4 11.4 MB)

(MP4 4.6 MB)

## Data Availability

No datasets were generated or analysed during the current study.

## Footnotes

1 http://omniweb.gsfc.nasa.gov.

2 Cell- or plume-like supergranular structure is also present within coronal holes, both in Fe ix 17.1 nm and in Fe xii 19.3 nm; see Figure 3 in Wang (2020), Alzate, Di Matteo, and Higginson (2025). The latter authors have likewise noted the role of such short-lived cells in expanding or shrinking the hole boundaries.

3 The PFSS extrapolation is expected to give only qualitative agreement with the observed hole boundaries, with the largest source of error likely to be the photospheric field measurements, which often do not adequately capture the lowest-order multipoles that determine the large-scale coronal field (see, e.g., Wang, Ulrich, and Harvey 2022; Wang and Samara 2024).
